# A practice-based approach to teaching antimicrobial therapy using artificial intelligence and gamified learning

**DOI:** 10.1093/jacamr/dlae099

**Published:** 2024-07-06

**Authors:** Sebastian Driesnack, Fabian Rücker, Nadine Dietze-Jergus, Alexander Bondarenko, Mathias W Pletz, Adrian Viehweger

**Affiliations:** Institute for Medical Microbiology and Virology, University of Leipzig Medical Center, Liebigstraße 21, 04103 Leipzig, Germany; Institute for Medical Microbiology and Virology, University of Leipzig Medical Center, Liebigstraße 21, 04103 Leipzig, Germany; Institute for Medical Microbiology and Virology, University of Leipzig Medical Center, Liebigstraße 21, 04103 Leipzig, Germany; Institute for Medical Microbiology and Virology, University of Leipzig Medical Center, Liebigstraße 21, 04103 Leipzig, Germany; Institute for Infectious Diseases and Infection Control, Jena University Hospital, Am Klinikum 1, 07747 Jena, Germany; Institute for Medical Microbiology and Virology, University of Leipzig Medical Center, Liebigstraße 21, 04103 Leipzig, Germany

## Abstract

**Objectives:**

Scalable teaching through apps and artificial intelligence (AI) is of rising interest in academic practice. We focused on how medical students could benefit from this trend in learning antibiotic stewardship (ABS). Our study evaluated the impact of gamified learning on factual knowledge and uncertainty in antibiotic prescription. We also assessed an opportunity for AI-empowered evaluation of freeform answers.

**Methods:**

We offered four short courses focusing on ABS, with 46 participating medical students who self-selected themselves into the elective course. Course size was limited by the faculty. At the start of the course, students were given a questionnaire about microbiology, infectious diseases, pharmacy and qualitative questions regarding their proficiency of selecting antibiotics for therapy. Students were followed up with the same questionnaire for up to 12 months. We selected popular game mechanics with commonly known rules for teaching and an AI for evaluating freeform questions.

**Results:**

The number of correctly answered questions improved significantly for three topics asked in the introductory examination, as did the self-assessed safety of prescribing antibiotics. The AI-based review of freeform answers was found to be capable of revealing students’ learning gaps and identifying topics in which students needed further teaching.

**Conclusions:**

We showed how an interdisciplinary short course on ABS featuring gamified learning and AI could substantially improve learning. Even though large language models are a relatively new technology that sometimes fails to produce the anticipated results, they are a possible first step in scaling a tutor-based teaching approach in ABS.

## Background

Antimicrobial resistance (AMR) results in difficult-to-treat infections, making the WHO declare it a public health threat.^1^ Counteracting AMR is attempted by teaching rational treatment principles, also called ‘antibiotic stewardship’ (ABS). However, teaching ABS is currently challenging in the German medical studies curriculum since the subject combines various disciplines, such as microbiology, pharmacology, infectious diseases and hospital hygiene. These are scattered over many semesters with little mutual reference or interconnection.

Dedicated tutoring is an appropriate teaching format primarily because of its considerable effect on learning.^2^ Since many medical disciplines treat infections, ABS teaching needs to scale beyond a small group of students. Self-tutoring is scalable, with ‘scalable’ meaning a teaching approach reaching many students. The most traditional self-tutoring medium—books—‘don’t work’ by missing interaction, resulting in the reader’s ignorance of their omissions.^3,4^ Also, scalable are massively open online courses (MOOCs), aiming at large audiences by digitizing traditional media. Since MOOC completion rates are typically 2%–10%,^5,6^ their effectiveness remains suboptimal,^7^ which can be assumed true for ABS courses of the WHO, CDC or Harvard’s ‘EdX’.

Digitized lookup tables (e.g. house-internal guidelines^8,9^) are commonly used. Decisions therein remain ‘black boxes’ and cannot be generalized to new situations.

## Objectives

We hypothesize that a dedicated ABS teaching format can achieve deep knowledge for a large audience. Supporting factors of meta-learning include habit formation, which can be achieved through gamified elements,^10–14^ and methods for long-term persistence of learned facts, called ‘mnemonic media’^15^ an example of which is spaced repetition learning cards. Therefore, this work aimed to assess whether games were a proper teaching method of ABS, improve practical ABS knowledge in student participants and trial AI-based evaluation of student responses. Our work contributes insights into the usefulness of using simple games when teaching. While most current approaches use analogue board or card games with complex rules, we chose games with simple mechanics already known to users, such as pairs.^16^

Additionally, with recent developments in artificial intelligence (AI), namely large language models (LLMs) such as OpenAI’s ‘ChatGPT’,^17,18^ new educational experiments are possible. We reasoned that tutoring would become scalable if AI could be used in chat, like Khan Academy's AI tutor Khanmigo.^19^ This personal AI addresses school children’s learning of mathematics. In an academic context, Ruan *et al*.^20^ use an AI tutor for language learning. Our work shows how AI can facilitate student evaluation and feedback. This allows us to identify knowledge gaps more precisely, which is the basis for individual tutoring.

## Methods

### Course participants

Four short courses focusing on ABS took place at the Institute of Medical Microbiology and Virology, University of Leipzig Medical Center, from July 2022 to February 2024. Forty-six students in their fourth year of studying medicine participated. They self-enrolled voluntarily through the faculty’s website. Each course comprised 27 h of teaching, split into 12 90-min sessions and additional hours of self-study. The faculty determined the time allocation in line with their guidelines for elective courses. The course content was based on the German Medical Association’s curriculum for further training of doctors in ABS.

### Design principles

We constructed the course by integrating the specific requirements of the subject matter. Since applying theoretical knowledge in practice is essential for knowledge internalization, we selected a flipped classroom model, in which students read new material independently and were encouraged to reflect on it.^21^ Shared time was used to practice treatment and case studies.^22^

Instead of directly developing a scalable solution, we first sought to optimize teaching success. Once this was observed, we investigated how it could be scaled. Since many course situations require feedback and adaptation of material to students, we sought to design AI in the form of LLMs in mind, mainly in a reflective role, providing feedback and guidance on course material.

### Implementation

Two professional microbiologists wrote materials, and a board-certified consultant for medical microbiology and virology and a reviewer from an external hospital reviewed them. Questions and case discussions reflected everyday clinical practice. We recorded short introductory podcasts for each lesson to guide and supplement students’ knowledge of infectious diseases and provided further texts about each week’s subject. This way, students could prepare before in-person meetings, leaving room for shared practice, as was the goal in a flipped classroom.^22^ Our course design demanded continuous practice. To enable learners, we implemented several practice elements in a mobile app, as all students had smartphone access. Regarding interface design, we followed standard practices (e.g. navigation gestures).^23,24^ The app was made available for Android and Apple devices.

Practice occurred primarily through flashcards and games. Students reviewed Flashcards with increasing intervals between repetitions (‘spaced repetition’). This ‘spacing effect’ is a robust phenomenon.^25,26^

**Figure 1.**
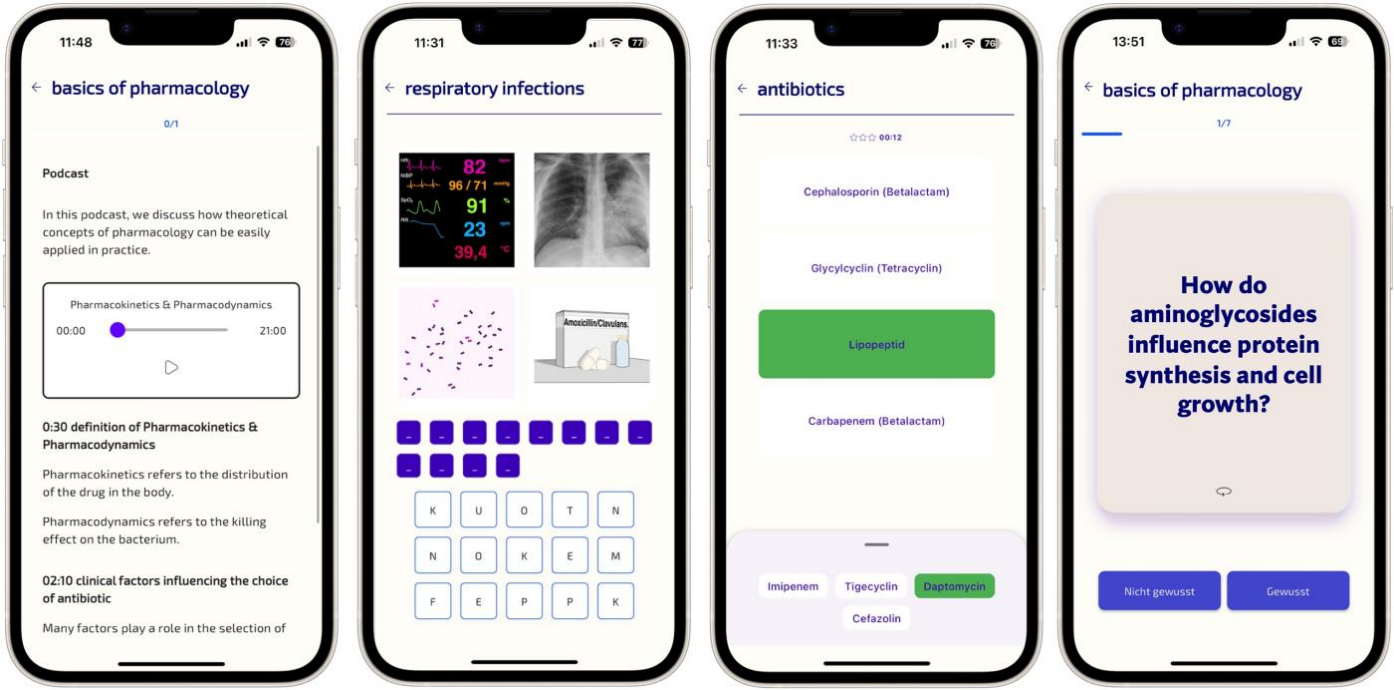
(From left to right) Podcast and additional material for students to prepare for the next lesson in a flipped classroom format. Game ‘4 pics 1 word’, where a word corresponding to four images shown should be guessed. Game ‘speed matching’, where pairs of matching concepts should be found under a time constraint. Flashcards are used with a spaced repetition schedule to support long-term memorization.

We used known prototypes for games, such as pairs, battleship, ‘4 pics one word’ and ‘speed matching’, where pairs of matching concepts should be identified under a time constraint, e.g. *E. coli* and Gram-negative (Figure 1). This example shows that we adapted known game mechanics to the infectious disease domain. Case studies were also practiced, and students were presented with anonymized, representative microbiological records from real patients. Cases were discussed during in-classroom sessions and in a chat format as homework in the app.

During the learning sessions, students also answered complex, freeform questions. Later, an LLM evaluated students’ answers, providing specific feedback and offering a general assessment of content difficulty and student ability.

### Example in-classroom session

While each in-classroom session was performed differently, many lessons shared a similar format. The typical in-classroom session started with gamified learning. Students formed groups of three and were given decks of cards from the game pairs. Cards showed, e.g. the micromorphology of pathogens or their names. Students started turning cards in search of pairs. The remaining time was aimed at higher-order thinking and acquiring skills in selecting antibiotics for therapy. Several clinical cases were discussed and supplemented with facts. For example, when discussing respiratory tract infections early in the course, the oropharyngeal resident flora was taught as a potential source for pathogens. Students could access supplementary in-app material (available as Supplementary data at *JAC-AMR* Online), which was dynamically provided after lessons.

### Evaluation

In all two-group comparisons (e.g. pre- and post-course knowledge about antibiotic treatment), we used a two-sided *t*-test with a threshold of *P* < 0.001 to accept the null hypothesis. In the factual assessment, we employed multiple-choice (MC) questions, while we measured responses to subjective, ordinal items (awareness, ability, proficiency) on a Likert scale (1 … 5), or, when more granularity was desired, a visual analogue scale (0 … 100). For the analysis of answers to freeform questions, we employed a LLM, namely ‘gpt-4-0125-preview’ (temperature = 0, OpenAI). To perform function calling for structured data generation, such as answer scores, we used the ‘instructor’ library (v0.4.5, Github-Website/jxnl/instructor). LLM calls were performed to promote consistent generations with the temperature set to zero. The alignment of LLM-generated and human feedback was quantified as interobserver agreement using Cohen’s kappa. We followed Landis and Koch for interpretation.^27^ To check for proportional bias, Bland–Altman plots were used.^28^

## Results

### Substantial and sustained improvement in factual knowledge after the course

Forty-six German students participated in our study, 15 males and 31 females. The median age of the participants was 24 years (range 22–34). Our questionnaire contained 18 MC questions about bacteria (*n* = 6), antibiotics (*n* = 5) and infectious diseases (*n* = 8; see supplement for the questionnaire) to assess student progress over the course. All 46 participants answered the same questions before and after the course. Because we reused these questions multiple times, students remained blinded to the correct answers.

**Figure 2.**
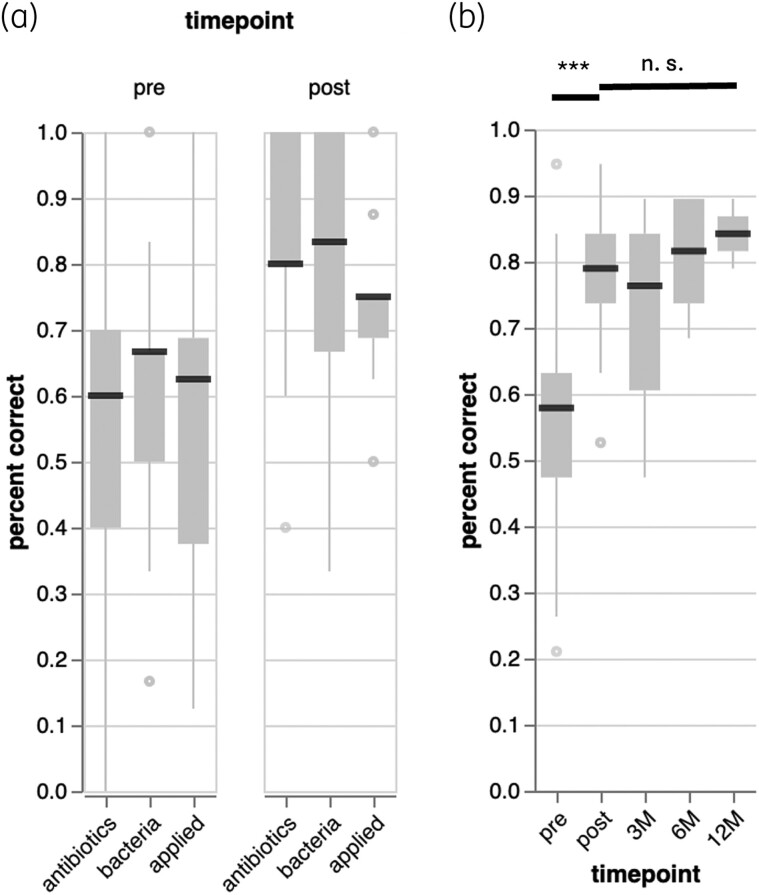
Substantial increases in knowledge over the course and knowledge persistence for up to 1 year. (a) A comparison of answers to 18 factual questions related to three topics (antibiotics, bacteria and ‘applied’ infectious diseases) before (‘pre’) and after (‘post’) the course revealed a significant improvement in all topics. (b) This knowledge persists for up to one year, with no significant (n.s.) decline in correctly answered questions when comparing the ‘post’ assessment to all subsequent time points.

We identified significant knowledge gaps within all three topics (Figure 2a). Overall, 57.22% of questions were answered correctly before the course, with wide variance among individual students (SD = 13.72%), suggesting heterogeneous skills. After the course, significantly more questions were answered correctly—for bacteria (mean = 81.40%, *P* < 0.001), antibiotics (mean = 83.26%, *P* < 0.001) and infectious diseases (mean = 74.42%, *P* < 0.001). Variance among students remained high (SD = 15%).

To assess knowledge retention and long-term memory, we followed up with students 3, 6 and 12 months after the end of the course, reasking the same questions as before. The number of respondents declined with time; at 3 months, 18 students responded (36.96%), six after 6 months (13.04%) and two after 12 months (4.35%). Despite this high dropout and putative dropout bias, the submitted answers suggest that knowledge gained persists for up to 1 year and likely beyond, as no downward trend was detected (Figure 2b).

### Reduction in uncertainty when selecting antimicrobial therapies

Right at the beginning, at the end and 3, 6 and 12 months after the course, we asked 15 qualitative questions about the students’ awareness of AMR as a significant problem in health care, their ability to address this problem and how proficient and secure they felt when selecting antibiotics for therapy.

Most students were keenly aware of the problem. For example, they estimated that approximately half of all antibiotics were prescribed unnecessarily (mean = 54.72, SD = 18.91), and on a visual analogue scale (VAS, 0 … 100), students reported on average 74.34 (SD = 19.30) for how concerned they were regarding AMR. One student reported during the course, ‘While we are told that AMR is a problem all the time and across multiple disciplines, we are never told what to do, practically speaking’. Many others shared versions of this viewpoint. The potential ability to address the challenge of AMR was strong; for example, on average, students opined that collective action could thwart the emergence and spread of AMR with 87.15 points (SD = 12.56) on a visual analogue scale from 0 to 100. Comparing awareness and ability-related items before and after the course, we found no significant difference (*P* > 0.01 for all items).

**Figure 3.**
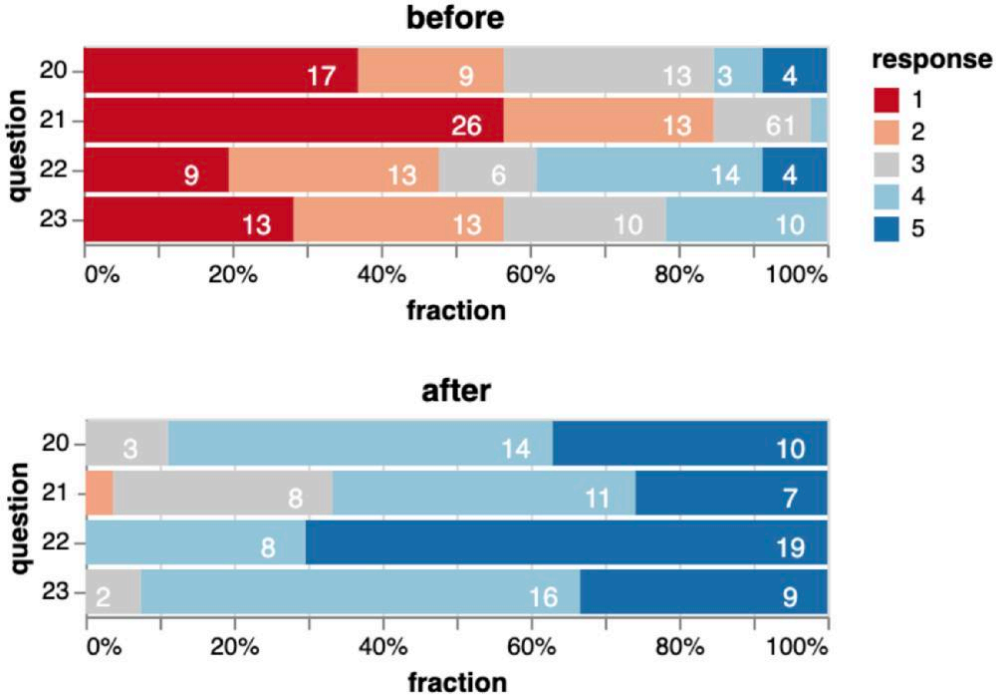
Subjective confidence rating of 46 students regarding antibiotic therapy initiation before (upper panel) and after (lower panel) the course. Rating was performed on a Likert scale from 1 (‘no agreement’) to 5 (‘full agreement’). A substantial improvement can be observed across all questionnaire items in this block (blue is better, colour number 5). While all 46 students performed the ‘before’ questionnaire, only 27 completed the evaluation ‘after’ the course.

However, students felt the course substantially improved their confidence when selecting antibiotics (Figure 3). For example, asked to estimate their ability to choose an empirical antibiotic treatment, their assessment changed from 1.61 pre-course (SD = 0.80, Likert scale from 1 to 5, where 1 means ‘no agreement’ and 5 means ‘full agreement’) to 3.89 afterward (SD = 0.85, *P* < 0.01).

### AI-based freeform answer reviews can reveal learning gaps

In addition to MC questions, we designed ten freeform questions containing short use case descriptions (e.g. a hypothetical patient who shows symptoms and has accompanying conditions) and asked to find a solution (e.g. a treatment programme). While MC questions allow automated assessment and capture factual errors well, they do not reveal erroneous reasoning or more complex mistakes.^29^ Freeform answers are more informative; however, their evaluation is laborious. Thus, we asked whether LLMs can be used to automate student feedback on unstructured assessments such as freeform questions and whether the LLM’s assessment was consistent with human evaluation of the same questions.

We asked 18 students ten freeform questions, which required 180 feedback responses. Although questions usually required multiple facts to be answered, our objective was to elicit understanding. For example, we asked: ‘While gentamicin is given once daily, flucloxacillin has to be dosed up to six times a day. Why?’. For each triple of a freeform question, expert answer and student response, we used GPT-4 to generate two assessment items: (i) specific feedback on how well the student answer aligns with the expert answer and (ii) topics that the student answer reveals a knowledge gap in, which the student could later revise or the tutor could use to improve teaching. Two human reviewers assessed the generated text to evaluate the quality of LLM-based feedback. On specific feedback, they rated whether the LLM-based assessment would be helpful to students, and on the extracted knowledge gap topics, whether they pointed into valuable directions for future studies. Furthermore, we used GPT-4 to rate the factual correctness of student answers. We used the evaluation template designed by OpenAI that evaluates the factuality of target texts (student answers) and the extent to which factual statements overlap with the facts in the expert answer.^30^ GPT-4 assigned one out of five categorical rates to the student’s answer. For example, ‘A’ indicates that the student’s answer is a subset of the expert answer, meaning that some of the facts from the expert answer were reproduced, but not all. ‘D’ indicates disagreement between student and expert answer. To add granularity, GPT-4 returned the percentage of how much the question was answered correctly. The two human reviewers performed the same assessment on a subset of student answers (*n* = 50) and were blinded to the LLM-generated evaluation.

On average, the human reviewers assessed 92% (*n* = 46) of the LLM feedback responses as helpful (Cohen’s kappa = 0.7, substantial agreement). None of the generated feedback introduced incorrect information (‘hallucinations’).

The LLM assessed no answers as entirely aligned with the expert answer (‘C’), but 16 of 180 student answers (8.89%) were classified as a subset (‘A’) and 15.56% as a superset (‘B’) of the expert answer. Only 1.11% were classified as ‘E’, i.e. the answers addressed the question correctly but in a way unexpected by the expert. Unsurprisingly, 74.44% of answers were assessed as ‘D’, indicating at least some disagreement with the expert answer. Human reviewers showed fair-to-moderate agreement with the LLM assessment on the class labels A–E (Cohen’s kappa was 0.36 and 0.42, respectively). However, even among human reviewers, agreement was only moderate (kappa = 0.56). When limiting the class labels to D and ‘not D’, akin to a true/false label, the agreement between LLM and human reviewers was moderate (kappa increased to 0.41 and 0.6, between LLM and each human reviewer, respectively). Using the LLM-assigned score, on average, 63.06% (SD = 18.41%) of each question agreed with the expert answer. Agreement between LLM and human reviewers on these scores was surprisingly consistent, as assessed using 95% CIs of the mean score differences (human 1: 14.4 ± 21.93%, human 2: 23.7 ± 28.34%), and differences were not significant (*P* > 0.01). We observed no proportional bias (Bland–Altman plot, data not shown).

**Figure 4.**
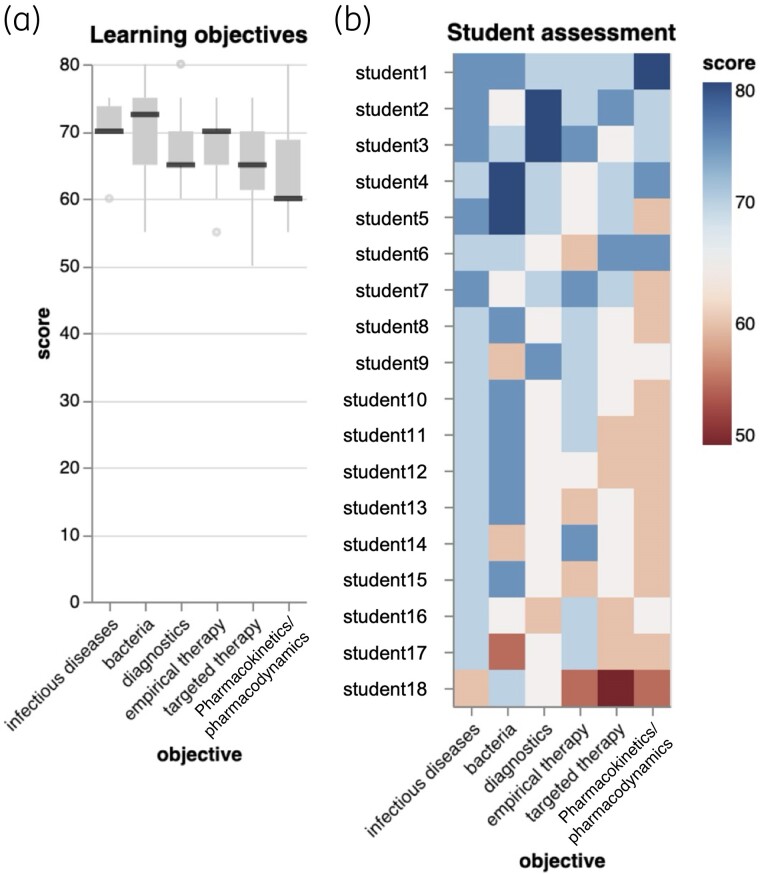
Synthesis task for the LLM to reason about learning topics instead of individual exam items. Note that the figures show the LLM-generated evaluation; for its validation by human reviewers, please refer to the main text. (a) Five learning topics were formulated for the course. For each student and objective, the combined LLM feedback across all freeform questions for that student was combined and given to the LLM to reason over. The aim was to estimate the probability that the student would be able to answer new questions sourced from the learning objective (‘score’). Since LLMs do not compute an actual probability, this is more of a framing to extract a quantitative metric from the model; in the figures, it is called ‘score’ instead of probability to avoid confusion. When these scores are aggregated, topics can be ranked by difficulty. Pharmacokinetics and pharmacodynamics appear to be the most challenging in the course. For clarity, note that the *y*-axis has been truncated to 80%. (b) These data can also be used to create student profiles, ranking them on their ability across learning topics. Individual students can then be targeted for additional learning support.

Having shown the feasibility of LLM-based assessment of individual test items, we asked whether the LLM could be used to evaluate overall learning success. Such a report could inform tutors about difficulties of specific learning topics and students’ learning progress. We formulated five learning topics we wanted students to learn about during the course: infectious diseases, bacterial properties, microbiological diagnostics, pharmacokinetics and pharmacodynamics and empirical and targeted antimicrobial therapy. We then employed the LLM to estimate how likely students were to answer a hypothetical question from this learning objective, given the feedback they received on each freeform question. The LLM generated a ‘score’, one for each student and learning topic, which could now be aggregated across topics as an indicator of the topic’s difficulty. A teacher could focus, e.g. on the most challenging topic (Figure 4a). We could also rank students, identifying those who struggled and might need more attention (Figure 4b). The ranking aligned with our experience teaching the course and getting to know students and their abilities.

## Discussion

We showed how an interdisciplinary short course on ABS could improve learning. We also integrated LLMs into the course, a first step in scaling the tutor-based approach.

The cumulative number of students was small (46 total). We assume that the observed increase in factual knowledge and increased security in choosing treatments will generalize to other populations, but there is a selection bias in the voluntary sign-up for a course on antibiotics and students might, therefore, be more motivated about the topic than average. Additionally, most did not respond to our questionnaire during follow-up, introducing follow-up bias and resulting in small sample sizes, especially at 6- and 12-month follow-ups.

Including gamification received positive (informal) feedback. However, while gamification and gamified learning have been described as motivational,^11^ there is doubt about the long-term benefits.^31^ We see optimistic data and will continue the gamified approach in future courses.

Attending was mandatory as part of their studies. This forced participation might have been the main reason for completing the course; however, our experience of talking to the students informally does not support this conclusion.

Interestingly, LLM-based feedback on freeform answers was assessed to be reasonably consistent with human reviewers. Ninety-two percent of the generated feedback responses were evaluated to be helpful to the student receiving it. The success of the per-item assessments led us to use the LLM to generalize the feedback and inform about question difficulty and student learning success. LLM-based feedback could thus adaptively match course content to a student’s needs in future work. But in their infancy, such feedback and evaluation systems have been described as accurate and valuable by Hooda *et al.*,^32^ mainly in non-medical fields like mathematics. We confirm that LLM-based automated assessments are possible and congruent with human evaluation. However, the limited sample size warrants further validation of the system’s robustness in future experiments. If sufficient calibration can be achieved with human reviewers, there might not be an ongoing need for human supervision of individual assessments.

Last, LLMs are a relatively new technology that sometimes fails to produce the anticipated results. Furthermore, their output still depends on careful, prompt engineering. However, if the current trajectory of improvement holds, LLMs will become a valuable teaching tool, potentially allowing a much more dynamic and adaptive way to instruct.

Rational use of antibiotics is required to curb increasing AMR rates globally. One main lever to achieve this is an improved teaching of ABS. We introduced a course concept that is very effective at transmitting this knowledge and can potentially be scaled to many participants using modern technologies.

## Supplementary Material

dlae099_Supplementary_Data

## Data Availability

All data and code have been committed to a dedicated repository without access limitations (Github website/phiweger/course).

## Supplementary data

Questionnaire in German is available as Supplementary data at *JAC-AMR* Online.

## References

[1] Antimicrobial Resistance Collaborators . Global burden of bacterial antimicrobial resistance in 2019: a systematic analysis. Lancet 2022; 399: 629–55. 10.1016/S0140-6736(21)02724-0 35065702 PMC8841637

[2] Bloom BS . The 2 sigma problem: the search for methods of group instruction as effective as one-to-one tutoring. Educ Res 1984; 13: 4–16. 10.2307/1175554

[3] Matuschak A . “Why books don't work”. San Francisco, 2019. https://andymatuschak.org/books

[4] Bergman A . Response to: Why books don’t work.

[5] Duncan A, Premnazeer M, Sithamparanathan G. Massive open online course adoption amongst newly graduated health care providers. Adv Health Sci Educ Theory Pract 2022; 27: 919–30. 10.1007/s10459-022-10113-x 35389153 PMC8988909

[6] Reich J . Failure to Disrupt: Why Technology Alone Can’t Transform Education. Harvard University Press, 2020.

[7] Kizilcec RF, Reich J, Yeomans M et al Scaling up behavioral science interventions in online education. Proc Natl Acad Sci U S A 2020; 117: 14900–5. 10.1073/pnas.1921417117 32541050 PMC7334459

[8] Schönherr SG, Wendt S, Ranft D et al Assessing the impact of institution-specific guidelines for antimicrobials on doctors’ prescribing behavior at a German tertiary-care center and the additional benefits of providing a mobile application. PLoS One 2020; 15: e0241642. 10.1371/journal.pone.0241642 33141858 PMC7608892

[9] Schönherr SG, Ranft D, Lippmann N et al Changes in antibiotic consumption, AMR and clostridioides difficile infections in a large tertiary-care center following the implementation of institution-specific guidelines for antimicrobial therapy: a nine-year interrupted time series study. PLoS One 2021; 16: e0258690. 10.1371/journal.pone.0258690 34648594 PMC8516227

[10] Subhash S, Cudney EA. Gamified learning in higher education: a systematic review of the literature. Comput Human Behav 2018; 87: 192–206. 10.1016/j.chb.2018.05.028

[11] Ishizuka K, Shikino K, Kasai H et al The influence of gamification on medical students’ diagnostic decision making and awareness of medical cost: a mixed-method study. BMC Med Educ 2023; 23: 813. 10.1186/s12909-023-04808-x 37898743 PMC10613361

[12] Gené OB, Núñez MM, Blanco ÁF. Gamification in MOOC: challenges, opportunities and proposals for advancing MOOC model. *Proceedings of the Second International Conference on Technological Ecosystems for Enhancing Multiculturality Salamanca, Spain, 2014*. 215–20.

[13] Buckley P, Doyle E. Gamification and student motivation. Interact Learn Environ 2016; 24: 1162–75. 10.1080/10494820.2014.964263

[14] Sailer M, Homner L. The gamification of learning: a meta-analysis. Educ Psychol Rev 2020; 32: 77–112. 10.1007/s10648-019-09498-w

[15] Matuschak A, Nielsen M. How can we develop transformative tools for thought? San Francisco, 2019. https://numinous.productions/ttft

[16] Nowbuth AA, Asombang AW, Alaboud K et al Gamification as an educational tool to address antimicrobial resistance: a systematic review. JAC Antimicrob Resist 2023; 5: dlad130. 10.1093/jacamr/dlad130 38089458 PMC10712719

[17] OpenAI . GPT-4 Technical Report. 2023; *arXiv [cs.CL]*.

[18] Minaee S, Mikolov T, Nikzad N et al Large Language Models: A Survey. 2024; *arXiv [cs.CL]*.

[19] Chen B, Zhu X, Díaz del Castillo HF. Integrating generative AI in knowledge building. Comput Educ Artif Intell 2023; 5: 100184. 10.1016/j.caeai.2023.100184

[20] Ruan S, Jiang L, Xu Q et al Englishbot: an AI-powered conversational system for second language learning. *Proceedings of the 26th International Conference on Intelligent User Interfaces, Station, Texas, 2021*. 434–44.

[21] Moon J . Using reflective learning to improve the impact of short courses and workshops. J Contin Educ Health Prof 2004; 24: 4–11. 10.1002/chp.1340240103 15069907

[22] Betihavas V, Bridgman H, Kornhaber R et al The evidence for ‘flipping out’: a systematic review of the flipped classroom in nursing education. Nurse Educ Today 2016; 38: 15–21. 10.1016/j.nedt.2015.12.010 26804940

[23] Tanil CT, Yong MH. Mobile phones: the effect of its presence on learning and memory. PLoS One 2020; 15: e0219233. 10.1371/journal.pone.0219233 32790667 PMC7425970

[24] Giannakas F, Kambourakis G, Papasalouros A et al A critical review of 13 years of mobile game-based learning. Educ Technol Res Dev 2018; 66: 341–84. 10.1007/s11423-017-9552-z

[25] Yuan X . Evidence of the spacing effect and influences on perceptions of learning and science curricula. Cureus 2022; 14: e21201. 10.7759/cureus.21201 35047318 PMC8759977

[26] Kang SHK . Spaced repetition promotes efficient and effective learning. PIBBS J 2016; 3: 12–9. 10.1177/2372732215624708

[27] Landis JR, Koch GG. The measurement of observer agreement for categorical data. Biometrics 1977; 33: 159–74. 10.2307/2529310 843571

[28] Ranganathan P, Pramesh CS, Aggarwal R. Common pitfalls in statistical analysis: measures of agreement. Perspect Clin Res 2017; 8: 187–91. 10.4103/picr.PICR_123_17 29109937 PMC5654219

[29] Roediger HL, Marsh EJ. The positive and negative consequences of multiple-choice testing. J Exp Psychol Learn Mem Cogn 2005; 31: 1155–9. 10.1037/0278-7393.31.5.1155 16248758

[30] OpenAI evals factuality assessment prompt . https://github.com/openai/evals/blob/main/evals/registry/modelgraded/fact.yaml.

[31] Hamari J . Transforming homo economicus into homo ludens: a field experiment on gamification in a utilitarian peer-to-peer trading service. Electron Commer Res Appl 2013; 12: 236–45. 10.1016/j.elerap.2013.01.004

[32] Hooda M, Rana C, Dahiya O et al Artificial intelligence for assessment and feedback to enhance student success in higher education. Math Probl Eng 2022; 1: 5215722. 10.1155/2022/5215722

