# Supplementary material for: A practice-based approach to teaching antimicrobial therapy using artificial intelligence and gamified learning
Source: JAC Antimicrob Resist. 2024 Jul 6;6(4):dlae099. doi: 10.1093/jacamr/dlae099 (PMC11227228; doi:10.1093/jacamr/dlae099)
Supplement: dlae099_Supplementary_Data [file dlae099_supplementary_data.docx]

**Fragebogen im Rahmen des Wahlfaches ABS**

(Q8) Von einer “Antibiotika-Krise” zu sprechen ist übertrieben.

Trifft nicht zu . Trifft eher nicht zu . Unentschlossen . Trifft eher zu . Trifft zu

(Q9) Ich schätze, der Anteil unnötig verordneter Antibiotika beträgt … (Kreuz auf Linie platzieren)

0%
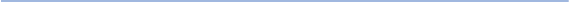
100%

—

(Q10) Inwieweit fühlst du dich verantwortlich, die Entstehung und Ausbreitung von Resistenzen zu verhindern?

überhaupt nicht
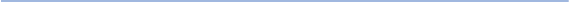
 absolut

—

(Q11) Wann, wenn überhaupt, denkst du, dass durch Antibiotikaresistenzen wieder Infektionen zu den häufigsten Todesursachen gehören?

schon jetzt
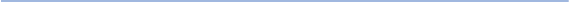
 > 100 Jahre/ nie

—

(Q12) Neu entdeckte Antibiotika werden jene ersetzen, die durch Resistenz unwirksam geworden sind.

Trifft nicht zu . Trifft eher nicht zu . Unentschlossen . Trifft eher zu . Trifft zu

—

(Q13) Wie besorgt bist du bezüglich des Themas “Antibiotikaresistenz”?

überhaupt nicht
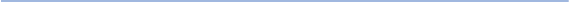
 absolut

—

(Q14) Ich werde aufgrund von Antibiotikaresistenz nicht mehr Zugang zu allen Therapien haben, die meine Eltern bekommen konnten.

Trifft nicht zu . Trifft eher nicht zu . Unentschlossen . Trifft eher zu . Trifft zu

—

(Q15) Inwieweit denkst du, kann dein eigenes Verhalten die Entstehung und Ausbreitung von Resistenzen verhindern?

überhaupt nicht
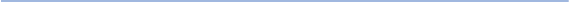
 absolut

(Q16) Inwieweit kann kollektives Verhalten die Entstehung und Ausbreitung von Resistenzen verhindern?

überhaupt nicht
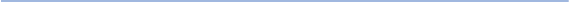
 absolut

—

(Q17) Der lokale Umgang mit Antibiotika hat globale Auswirkungen.

Trifft nicht zu . Trifft eher nicht zu . Unentschlossen . Trifft eher zu . Trifft zu

—

(Q18) Es lohnt sich **nicht**, der Resistenzentwicklung lokal entgegenzuwirken. Der Einsatz von Antibiotika in der Landwirtschaft und der mangelhafte Umgang mit den Substanzen in anderen Ländern heben alle eventuellen Fortschritte auf, um nur einige Beispiele zu nennen.

Trifft nicht zu . Trifft eher nicht zu . Unentschlossen . Trifft eher zu . Trifft zu

—

(Q19) Ich vermute, in meinem zukünftigen beruflichen Alltag oft die Hilfe von Fachexperten (Infektiologie, Mikrobiologie, Apotheke) in Anspruch nehmen müssen, um rationale Antibiosen zu verordnen.

Trifft nicht zu . Trifft eher nicht zu . Unentschlossen . Trifft eher zu . Trifft zu

—

(Q20) Ich gehe strukturiert vor bei der Auswahl von Antibiotika und kann bei Änderungen der Bedingungen sowohl gezielt eskalieren als auch deeskalieren.

Trifft nicht zu . Trifft eher nicht zu . Unentschlossen . Trifft eher zu . Trifft zu

—

(Q21) Ich fühle mich sicher bei der “kalkulierten” Antibiotikawahl, d.h. dem Verschreiben einer Substanz in Abwesenheit relevanter Informationen wie Infektionserreger und Resistogramm.

Trifft nicht zu . Trifft eher nicht zu . Unentschlossen . Trifft eher zu . Trifft zu

—

(Q22) Bei Fragen bezüglich der antiinfektiven Therapie kenne ich verlässliche Informationsquellen und weiß mir damit üblicherweise selbst zu helfen.

Trifft nicht zu . Trifft eher nicht zu . Unentschlossen . Trifft eher zu . Trifft zu

—

(Q23) Ich habe einen guten Überblick, welche Substanzen mir zur antiinfektiven Therapie zur Verfügung stehen.

Trifft nicht zu . Trifft eher nicht zu . Unentschlossen . Trifft eher zu . Trifft zu

—

(Q24) Ich habe schon einmal KI (z.B. ChatGPT) benutzt, um medizinische Themen zu lernen.

Trifft nicht zu . Trifft eher nicht zu . Unentschlossen . Trifft eher zu . Trifft zu

—

(Q25) Wenn ja, welche Erfahrungen hast du mit dem Einsatz von KI für diese Aufgabe (medizinische Themen zu lernen) gemacht?

(Q25.1) Ich habe Fragen zu einem Thema gestellt, mit dem ich nicht vertraut war.

Trifft nicht zu . Trifft eher nicht zu . Unentschlossen . Trifft eher zu . Trifft zu

(Q25.2) Ich habe KI gebeten, ein Thema zu erklären, das ich schon ein wenig kannte.

Trifft nicht zu . Trifft eher nicht zu . Unentschlossen . Trifft eher zu . Trifft zu

(Q25.3) Ich nutzte KI, um zu kontrollieren, ob mein Wissen zu einem Thema korrekt ist.

Trifft nicht zu . Trifft eher nicht zu . Unentschlossen . Trifft eher zu . Trifft zu

(Q25.4) Sonstiges (Freitext):

—

(Q26) Ich glaube, dass KI / LLMs / Chatbots für das medizinische Lernen eingesetzt werden können.

Trifft nicht zu . Trifft eher nicht zu . Unentschlossen . Trifft eher zu . Trifft zu

—

(Q27) Welche Bedenken hast du beim Einsatz von KI / LLMs / Chatbots zum Lernen in der Medizin (Freitext)?

—

(Q28) Was würdest du dir in einer idealen Welt von einem KI-/LLM-/Chatbot-System wünschen, das dir beim medizinischen Lernen helfen kann (Freitext)?

**Q24 Welcher der nachfolgenden Erreger stellt sich in der Gramfärbung als gram-negatives Stäbchen dar?**

A Staphylococcus aureus

B Streptococcus pneumoniae

C Streptococcus pyogenes

D Escherichia coli

E Corynebacterium diphtheriae

**Q25 Welches der nachfolgenden Medikamente gehört zu den Cephalosporinen?**

A Penicillin

B Ampicillin

C Imipenem

D Gentamicin

E Ceftazidim

**Q26 Mit welcher Substanz würden Sie eine Harnwegsinfektion mit Nachweis von E.coli kalkuliert behandeln?**

A Gentamicin

B Penicillin

C Cefotaxim

D Tigecyclin

E Vancomycin

**Q27 Wie stellt sich Pseudomonas aeruginosa in der Gramfärbung dar?**

A Gram-positive Kettenkokken

B Gram-positive Haufenkokken

C Gram-negative Diplokokken

D Gram-positive Stäbchen

E Gram-negative Stäbchen

**Q28 Welcher Substanzklasse wird Meropenem zugeordnet?**

A Cephalosporine

B Aminopenicilline

C Aminoglycoside

D Carbapeneme

E Fluorochinolone

**Q29 Welche kalkulierte antibiotische Therapie würden Sie bei einem Patienten mit nosokomialer Pneumonie beginnen?**

A Cefotaxim

B Levofloxacin

C Teicoplanin

D Piperacillin-Tazobactam

E Ampicillin-Sulbactam

**Q30 Sie sehen im mikroskopischen Bild eines Ohrabstriches gram-positive Diplokokken. Um welchen der nachfolgenden Erreger handelt es sich?**

A Neisseria meningitidis

B Listeria monocytogenes

C Staphylococcus aureus

D Streptococcus pneumoniae

E Streptococcus pyogenes

**Q31 Welches der nachfolgenden Antibiotika weist eine gute Liquorgängigkeit auf?**

A Ciprofloxacin

B Rifampicin

C Piperacillin/Tazobactam

D Cefotaxim

E Gentamicin

**Q32 Welches der nachfolgenden Medikamente setzen Sie zur kalkulierten Therapie einer ambulant erworbenen Pneumonie ein?**

A Meropenem

B Amikacin

C Ampicillin-Sulbactam

D Cotrimoxazol

E Moxifloxacin

**Q33 Für welche der nachfolgenden kalkulierten Antibiotikatherapien entscheiden Sie sich bei einer akuten Cholezystitis, die zunächst konservativ behandelt werden soll?**

A Cefotaxim und Metronidazol

B Vancomycin und Tigecyclin

C Teicoplanin und Gentamicin

D Ampicillin und Ciprofloxacin

E Piperacillin/Tazobactam und Metronidazol

**Q34 Wie stellt sich Haemophilus influenza in der Gramfärbung dar?**

A Gram-positive Kettenkokken

B Gram-positive Haufenkokken

C Gram-negative Diplokokken

D Gram-positive Stäbchen

E Gram-negative Stäbchen

**Q35 Ein wichtiger Erreger der Neugeborenen-Meningitis sind B-Streptokokken.
Woher stammen die Erreger typischerweise?**

A Gastrointestinaltrakt des Kindes

B physiologische Hautflora des Kindes

C physiologische Rachenflora des Kindes

D Vaginalflora der Mutter

E physiologische Rachenflora der Mutter

**Q36 Im Grampräparat eines intraoperativ gewonnenen Wundabstriches vom Unterschenkel sehen Sie gram-positive Haufenkokken. Klinisch handelt es sich um eine Phlegmone. Für welche kalkulierte Antibiotikatherapie entscheiden Sie sich?**

A Penicillin

B Imipenem

C Cefotaxim

D Clindamycin

E Ciprofloxacin

**Q37 Welcher der nachfolgenden Erreger ist am häufigsten ursächlich für Knochen- und Weichteilinfektionen?**

A Streptococcus pyogenes

B Enterococcus faecalis

C Corynebacterium aurimucosum

D Escherichia coli

E Staphylococcus aureus

**Q38 Welches der nachfolgenden Antibiotika wirkt bakteriostatisch?**

A Daptomycin

B Ertapenem

C Cefotaxim

D Gentamicin

E Clarithromycin

**Q39 Welches der folgenden Antibiotika gehört nicht in die Gruppe der β-Lactamantibiotika?**

A Ertapenem

B Daptomycin

C Cefotaxim

D Aztreonam

E Ceftazidim

**Q40 Anhand ihres Zellwandaufbaus kann man mittels Grammfärbung grampositive und gramnegative Bakterien unterscheiden. Was ist ursächlich für das unterschiedliche Anfärbeverhalten der Bakterien?**

A Die Anzahl der Penicillinbindeproteine

B Das Vorhandensein von Lipopolysacchariden

C Die Abgrenzung durch eine äußere Membran

D Unterschiede in der Dicke der Peptidoglycanschicht

E Die Durchlässigkeit von Porinkanälen

**Q41 Welche der nachfolgenden Aussagen zur Blutkulturdiagnostik ist zutreffend?**

A Die Entnahme von Blutkulturen gehört zur Standarddiagnostik und sollte bei jedem stationären Patienten durchgeführt werden

B Da das Blutentnahmevolumen möglichst gering sein sollte, wird empfohlen pro

Patient nur ein Flaschenpaar zu beimpfen

C Um die Untersuchungsergebnisse nicht zu verfälschen, sollten Blutkulturen bis

zum Versand ins Labor im Kühlschrank gelagert werden

D Die Transportzeit von Blutkulturflaschen spielt für die nachfolgende Diagnostik

keine entscheidende Rolle

E Mittels Blutkulturen können alle relevanten humanpathogenen bakteriellen Erreger

nachgewiesen werden

**Q42 Ein junger Patient wird appendektomiert. Als perioperative Antibiose erhält er ein Cephalosporin der Gruppe 2. Am nächsten Tag hat er Fieber und seine Bauchdecke ist gespannt. Gegen welchen Erreger hat die Antibiose sicher nicht gewirkt?**

A Enterococcus faecalis

B Staphylococcus aureus

C Streptococcus pneumoniae

D Escherichia coli

E Haemophilius influenzae
